# Effect of Subglottic Stenosis on Vocal Fold Vibration and Voice Production Using Fluid–Structure–Acoustics Interaction Simulation

**DOI:** 10.3390/app11031221

**Published:** 2021-01-29

**Authors:** Dariush Bodaghi, Qian Xue, Xudong Zheng, Scott Thomson

**Affiliations:** 1 Department of Mechanical Engineering, University of Maine, Orono, ME 04473, USA; 2 Department of Mechanical Engineering, Brigham Young University, Provo, UT 84602, USA

**Keywords:** subglottic stenosis, vocal fold, voice production, fluid–structure–acoustic interaction, hydrodynamic/acoustics splitting method, linearized perturbed compressible equation, computational fluid dynamics, flow resistance

## Abstract

An in-house 3D fluid–structure–acoustic interaction numerical solver was employed to investigate the effect of subglottic stenosis (SGS) on dynamics of glottal flow, vocal fold vibration and acoustics during voice production. The investigation focused on two SGS properties, including severity defined as the percentage of area reduction and location. The results show that SGS affects voice production only when its severity is beyond a threshold, which is at 75% for the glottal flow rate and acoustics, and at 90% for the vocal fold vibrations. Beyond the threshold, the flow rate, vocal fold vibration amplitude and vocal efficiency decrease rapidly with SGS severity, while the skewness quotient, vibration frequency, signal-to-noise ratio and vocal intensity decrease slightly, and the open quotient increases slightly. Changing the location of SGS shows no effect on the dynamics. Further analysis reveals that the effect of SGS on the dynamics is primarily due to its effect on the flow resistance in the entire airway, which is found to be related to the area ratio of glottis to SGS. Below the SGS severity of 75%, which corresponds to an area ratio of glottis to SGS of 0.1, changing the SGS severity only causes very small changes in the area ratio; therefore, its effect on the flow resistance and dynamics is very small. Beyond the SGS severity of 75%, increasing the SGS severity, leads to rapid increases of the area ratio, resulting in rapid changes in the flow resistance and dynamics.

## 1. Introduction

Subglottic stenosis (SGS) is a narrowing of the airway between the larynx and trachea. The most frequent cause of SGS is post-intubation injury in which edema and inflammation cause the formation of ulcer and granulation tissues [1,2]. SGS has also been reported in patients having congenital diseases or traumas, treated by tracheotomy method, etc. The narrowing due to SGS can affect both breathing and voice with the symptoms, including noisy breathing, shortness of breath, hoarse voice, and dysphonia [3-5].

The severity of SGS is generally categorized into four grades by the level of obstruction of the airway: Grade I, below 50% obstruction; Grade II, 51–70% obstruction; Grade III, 71–99% obstruction; and Grade IV, 100% obstruction [6]. Treatment is different depending on the severity. For lower grades (Grades I and II), little to no treatment may be needed. For higher grades (Grades III and IV), open airway reconstructive procedures may be needed to restore airway patency; however, voice changes before and after the procedures were reported in clinical studies [7-10]. For example, Ettema et al. [11] did a perceptual study on the voice change due to SGS and found that about 50% of the patients’ voices were moderately to extremely affected. More interestingly, their results revealed that the effect of SGS on voice was not correlated with its severity, because some patients with a higher grade SGS retained normal voice quality. However, no detailed qualitative or quantitative data were available for a systematic understanding of the underlying reason.

Voice production is driven by a fluid–structure interaction process in which the air forced from the lungs interacts with the vocal folds, to generate self-sustained vibration of the vocal folds. Vocal fold vibration, in turn, modulates the airflow into a pulsatile flow, to form the sound source. Voice quality is indirectly related to the dynamics of the glottal flow and vocal fold vibrations. To understand how SGS affects voice production, it is necessary to understand how SGS affects the dynamics of the glottal flow and vocal fold vibration.

Several studies used computational fluid dynamics models to evaluate the effect of the severity of SGS on airway pressure drop [12-15] and flow resistance [16,17] (defined as ΔP∕Q or $\Delta P∕Q^{2}$, in which ΔP is the pressure drop and Q is the mean flow rate) during respiration; however, the effect of SGS on vocal fold vibration was not studied. To the best of the authors’ knowledge, the only work focusing on the effect of SGS on vocal fold vibration was a study by Smith and Thomson [18]. In this work, an idealized SGS was incorporated into a two-dimensional airway and fluid–structure interaction simulations were performed, to examine the effect of SGS on vocal fold vibration. The severity of SGS was measured as the percentage of the width reduction of the airway and varied from 0% to 99%, to cover the possible pathological range. The study found no change in vocal fold vibration and glottal flow rate up to 60% of SGS severity. By increasing the SGS severity to 90%, a decrease in vocal fold vibration was observed. By further increasing the SGS severities, both vocal fold vibration and the flow rate amplitude were decreased. However, the decrease in the maximum flow declination rate (MFDR, defined as the maximum negative slope of the glottal flow rate waveform [19]) and fundamental frequency $\left(f_{0}\right)$ was observed only when the SGS severity was above 95%. The study also suggested that these effects were primarily related to the effect of SGS on flow resistance.

While the study provides quantitative measurements of the effect of SGS on vocal fold vibration, it is limited by the underlying two-dimensional (2D) assumption. It is unclear if the effects remain the same in three-dimensional (3D) geometries, and more importantly, how the effects are related to the 3D properties of SGS. In the current study, we used an in-house 3D fluid–structure–acoustic interaction numerical solver to quantify the effect of SGS on the dynamics of glottal flow and vocal fold vibration in an idealized 3D vocal tract model. Two SGS properties, the percentage of area occlusion and the location of the SGS, were varied to investigate the effect of these two properties on the glottal flow rate and its waveform; vocal fold vibration amplitude, primary vibratory modes, and voice acoustics were quantified. In the context below, the modeling method, including the numerical algorithms of the computational solver, geometric models, and simulation setup, is presented in Section 2; the results and discussions are presented in Section 3, and the conclusion is presented in Section 4.

## 2. Models

### 2.1. Numerical Algorithms

The glottal flow dynamics was governed by the three-dimensional, unsteady, incompressible Navier–Stokes equations, as shown below:

(1) $$\;\frac{\partial V_{i}}{\partial x_{i}}=0\;\frac{\partial V_{i}}{\partial t}+\frac{\partial V_{i}V_{j}}{\partial x_{j}}=-\frac{1}{\rho_{0}}\frac{\partial P}{\partial x_{i}}+v_{0}\frac{\partial^{2}V_{i}}{\partial x_{j}\partial x_{j}}$$

where $\rho_{0}$, V, P, and $v_{0}$ are incompressible flow density, velocity, pressure, and kinematic viscosity. The equations are solved on a Cartesian mesh. A sharp-interface immersed boundary method is used to treat complex and moving boundaries. The details of the solver, numerical schemes, and discretization can be found in Reference [20].

Linearized perturbed compressible equations (LPCE) based on the hydrodynamic/acoustic splitting method [21] were used to simulate vocal tract acoustics:

(2) $$\;\frac{\partial v_{i}^{\prime }}{\partial t}+\frac{\partial v_{i}^{\prime }V_{i}}{\partial x_{j}}+\frac{1}{\rho }\frac{\partial p^{\prime }}{\partial x_{j}}=0\;\frac{\partial p^{\prime }}{\partial t}+V_{i}\frac{\partial p^{\prime }}{\partial x_{i}}+\gamma P\frac{\partial v_{i}^{\prime }}{\partial x_{i}}+v_{i}^{\prime }\frac{\partial P}{\partial x_{i}}=-\frac{dP}{dt}$$

where $v_{i}^{\prime }$ and $p^{{}_{{}_{\prime }}}$ are the perturbed compressible velocity components and pressure, and γ is the ratio of specific heats. The equations are also solved on a Cartesian mesh with the immersed boundary method to treat the boundaries. In the computation, the incompressible flow is computed first, and the obtained flow solution is used to compute the perturbed compressible variables. The final total velocity and pressure variables are computed as follows:

(3) $$v_{i}=v_{i}^{\prime }+V_{i}\;\;p=p^{\prime }+P$$

where v and p are the total flow velocity and pressure. This method was successfully verified in simulating acoustics in human phonation [22]. Further details of the LPCE equations can be found in Reference [21].

The governing equations of the vocal fold dynamics are Navier equations, as shown below:

(4) $$\rho \frac{\partial^{2}d_{i}}{\partial t^{2}}=\frac{\partial \sigma_{ij}}{\partial x_{j}}+\rho f_{i}$$

where ρ, d, σ, and f are tissue density, displacement, stress, and body force per unit mass, respectively. The finite element method was used to solve Equation (4) [23].

The above three solvers are explicitly coupled through a Lagrangian interface, which is represented by triangular surface meshes. The incompressible flow solver is marched by one step with the existing deformed shape and velocities of the solid tissue as the boundary conditions. The perturbed compressible solver is then marched with the updated incompressible flow field, as well as the existing deformed shape and velocities of the solid tissue as the boundary conditions. The forces at the vocal fold surface are then calculated with the new incompressible flow pressure and the perturbed pressure. At last, the solid solver is marched by one step with the updated surface traction. The deformation and velocities on the solid grid are then transferred to the vocal fold surface, so that the fluid/solid interface can be updated. The details of the coupling can be found in Zheng et al. [23] and Jiang et al. [24].

### 2.2. Geometric Models

**Figure 1.**
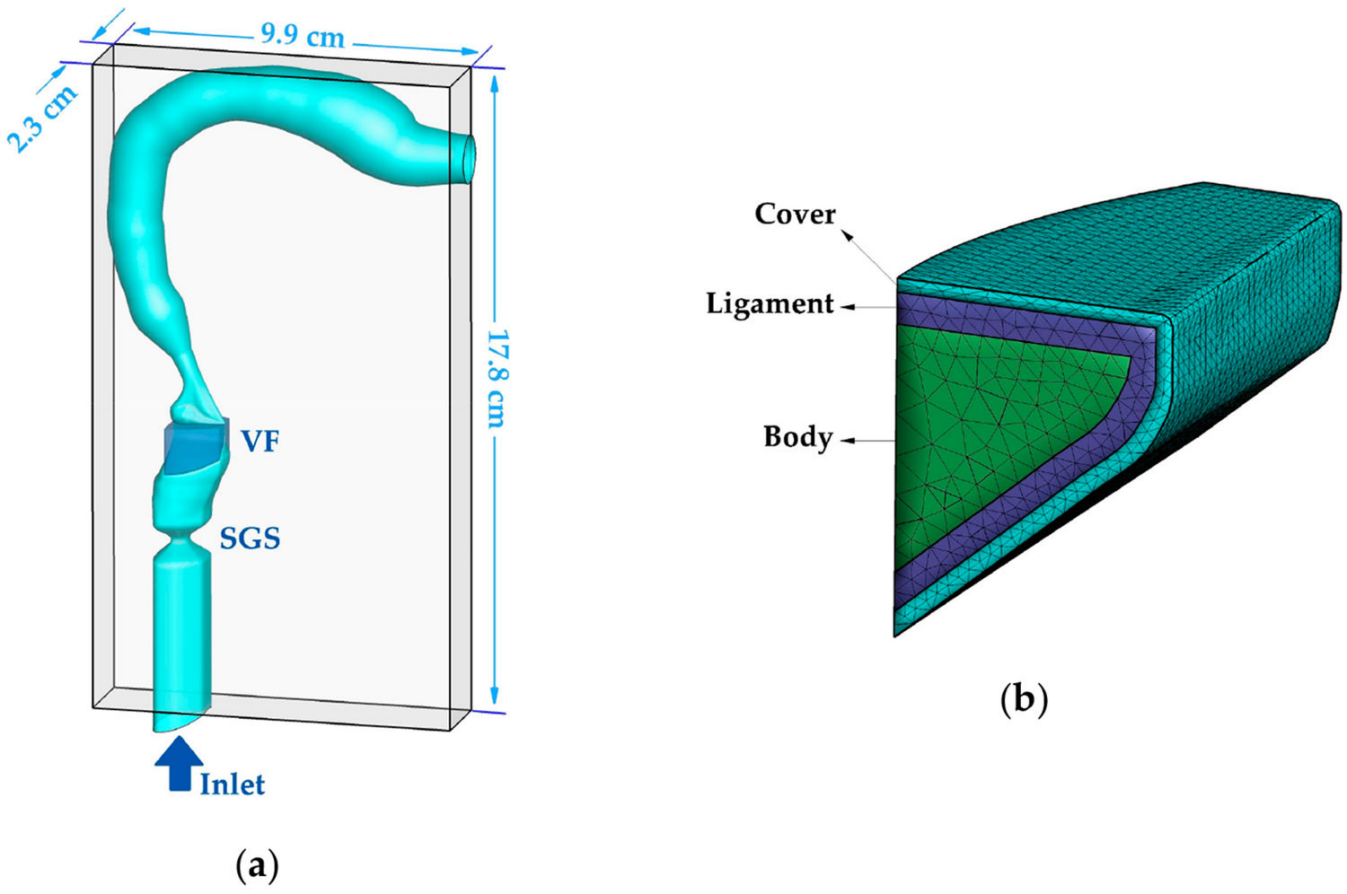
(**a**) The computational domain and geometry of the larynx, subglottic stenosis, vocal fold, and vocal tract. (**b**) The inner layers’ structure of the vocal fold.

Figure 1a shows the geometric models of the vocal folds, SGS, supraglottal tract, and subglottal tract, as well as the dimension of the computational domain. The configuration of the supraglottal airway was generated based on a neutral area function reported in Story [25], which is the mean area function of 12 vowels of a male speaker. According to another study by Story [26], the first and second natural frequencies (F1 and F2) of the mean area function are similar to those from a uniform tube, but higher frequencies are more subject-specific. The overall length of the supraglottal airway centerline is 17.4 cm. The geometries of the ventricle, false vocal folds, and subglottic tract were superimposed to the tract based on CT scans of a male subject larynx [27].

The geometry of the vocal fold (Figure 1b) was generated by using a mathematical model proposed by Titze and Talkin [28] that matches physiologically realistic key parameters and features (e.g., shape of medial surface, anterior–posterior shape variation, and the subglottal angle). The model assumes a three-layer structure of the vocal fold including the cover, ligament, and body, as shown in Figure 1b. The thickness of the cover and ligament layer was set as 0.5 and 1.1 mm, respectively, based on the average values of human measurement [29]. The vocal fold tissues were modeled as viscoelastic transversely isotropic materials. The material properties of each layer are listed in Table 1. These values were adopted from previous studies which showed how to reproduce typical vocal fold dynamics [24,30,31].

**Figure 2.**
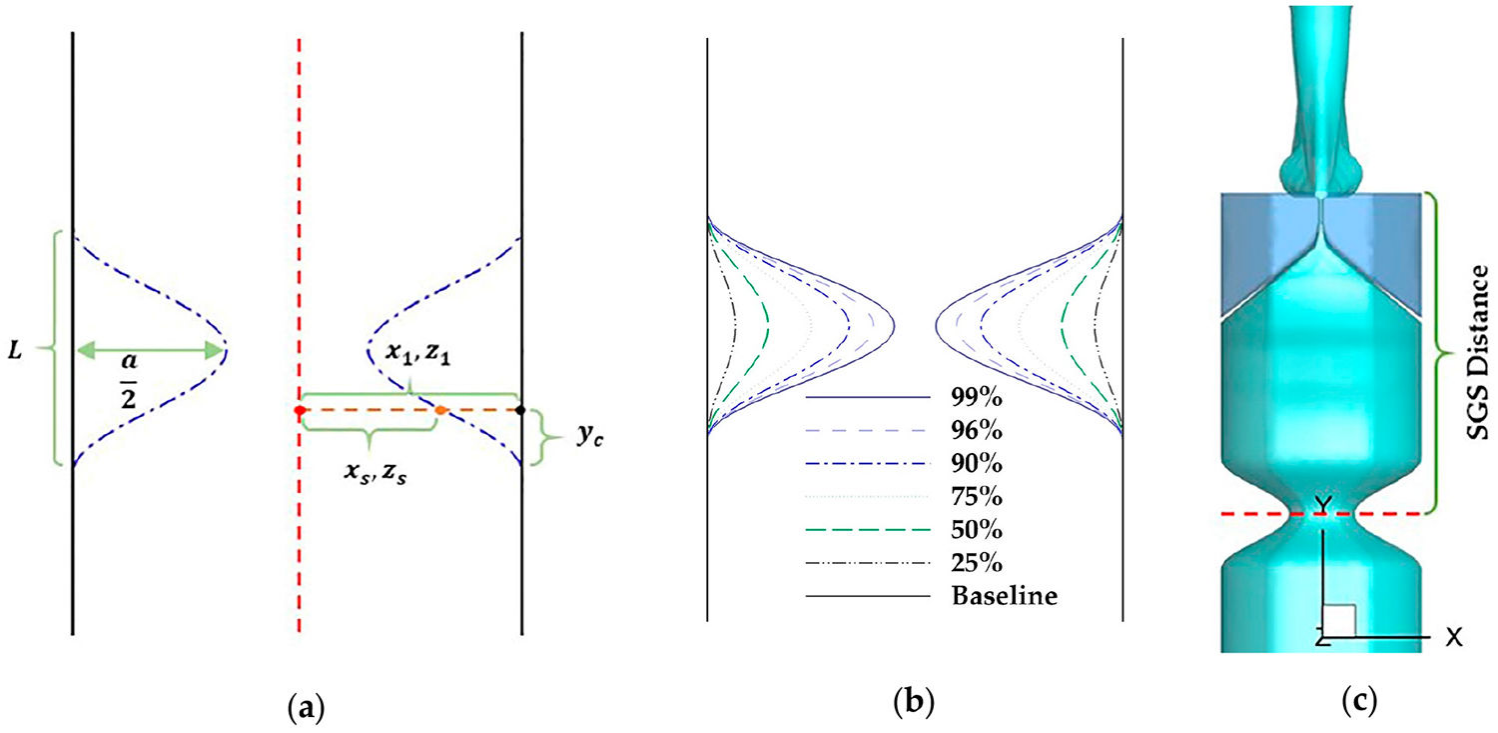
(**a**) Schematics of the cross-section of an idealized subglottic stenosis (SGS) geometry and the parameters used in Equation (5), to create the SGS. (**b**) The different values of SGS severity, which is defined as the percentage of area reduction due to SGS. (**c**) The SGS location, defined as the distance between the narrowest section of the SGS and superior surface of the vocal folds.

The subglottic tract was extended downward by 6.6 cm to incorporate the SGS. An idealized geometry of the SGS was created by using a cosine function (Figure 2a). Specifically, for a given surface point $\left(x_{1},y_{1},z_{1}\right)$ at the SGS region where $x_{1}$, $y_{1}$, and $z_{1}$ are the original coordinates of the point, the new coordinates $\left(x_{s},y_{s},z_{s}\right)$ of the point after adding the SGS were determined by using the equation below:

(5) $$x_{s}=x_{1}-x_{1}\times \frac{a}{2}\times \left(1-cos\left(2\pi \frac{y_{c}}{L}\right)\right)\;=z_{s}=z_{1}-z_{1}\times \frac{a}{2}\times \left(1-cos\left(2\pi \frac{y_{c}}{L}\right)\right)\;\;y_{s}=y_{1}$$

where L is the SGS length and was set as 1.0 cm, which is the most common SGS length [32]; $y_{c}$ is the distance of the lower bound of the SGS to the y component of the point $\left(y_{1}\right)$; and a is the percentage reduction of the hydraulic diameter of the airway cross-section due to the SGS.

In this study, the severity and location of SGS were both varied. The SGS severity was quantified by the percentage of area reduction due to SGS. Seven values were used, including 0% (baseline case), 25%, 50%, 75%, 90%, 96%, and 99%, by varying ‘‘a’’ in Equation (5) from 0 to 0.9 (Figure 2b). The SGS location was defined as the distance between the narrowest section of the SGS and the superior surface of the vocal folds (Figure 2c). Two values were used, 2.1 and 3.13 cm, which respectively correspond to the minimum and average values of 92 SGS patients reported in the past studies [32,33].

### 2.3. Computational Domain and Boundary Conditions

The entire geometric model was immersed into a 2.3 cm × 17.8 cm × 9.9 cm rectangular computational domain. The domain was discretized by a 64 × 256 × 92 Cartesian grid in the x, y, and z directions, respectively. A 0.8 kPa pressure drop was applied between the inlet and outlet to drive the flow. The total reflection boundary condition for acoustics was applied at the outlet. To eliminate the acoustic reflection at the inlet boundary, an anechoic zone was enforced in the LPCE solver to buffer the acoustics [34]. Vocal tract walls were treated as no-slip boundaries in the incompressible solver and hard wall boundary condition in the LPCE solver [35]. A traction boundary condition was applied at the fluid-structure interaction interface, while the lateral, anterior and posterior surfaces of the vocal folds were fixed in the solid solver. A penalty-method-based contact model was applied to prevent the penetration of the vocal folds. An artificial minimum gap of 0.2 mm was enforced between the vocal folds, which is necessary for the success of the flow solver. The vocal fold was discretized, using 28,997 tetrahedral elements. The time-steps of 1.149 × 10^−6^ s and 0.05 × 1.149 × 10^−6^ s were used in the incompressible and LPCE solvers, respectively, to satisfy their Courant–Friedrichs–Lewy condition of below 1. 256 processors were used to simulate 4.6 × 10^−2^ s on the XSEDE COMET cluster (Intel Xeon E5-2680v3 processors) which resulted in the computational cost of 161,000 CPU hours.

## 3. Results and Discussion

In this section, the results of the baseline case, including the dynamics of the glottal flow, vocal fold vibration, and acoustics, are first presented to demonstrate that the simulation represents typical human phonatory dynamics. Next, the changes of the dynamics with the varied SGS severity (area reduction) and location in the cases are quantified. The relationship between the quantities and SGS severity and locations is discussed. Finally, the underlying mechanism responsible for the relationships is analyzed.

### 3.1. Baseline Case Dynamics

#### 3.1.1. Glottal Flow Rate Waveform

**Figure 3.**
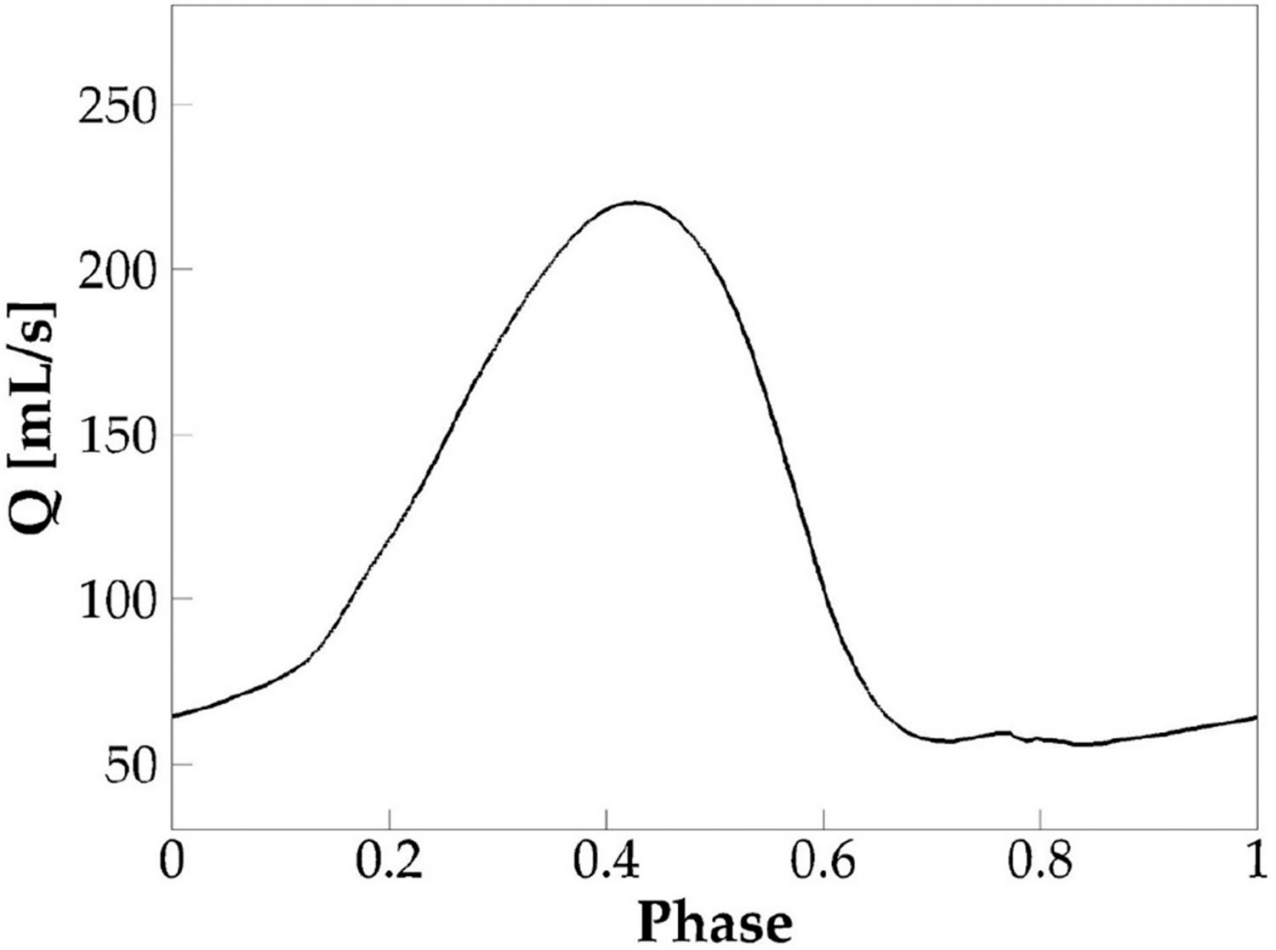
The phase-averaged flow rate over one vibration cycle in the baseline case.

The simulation was successfully carried out for eight vocal fold vibration cycles. Self-sustained vibration was established after the second cycle. Figure 3 shows the phase-averaged volumetric flow rate at the glottic exit based on the steady cycles. Phase-averaging is done by dividing each steady cycle into 1000 phases and calculating the average value at each phase over all the steady cycles. The flow rate shows a typical glottal flow rate waveform of human phonation which is characterized by a slower rising, faster dropping, and a lasting period of glottic closure. Notice that the flow rate did not drop to zero during glottic closure due to the artificial minimum glottic gap enforced in the simulation. Several typical voice quality related quantities were computed based on the waveform, including the $f_{0}$; the skewness quotient $\left(\tau_{s}\right)$, defined as the ratio of the duration of flow acceleration to flow deceleration; the peak glottal flow rate; the mean glottal flow rate; and the MFDR. The values are found within the typical physiological ranges of human phonation [31] (Table 2).

#### 3.1.2. Vocal Fold Dynamics

**Figure 4.**
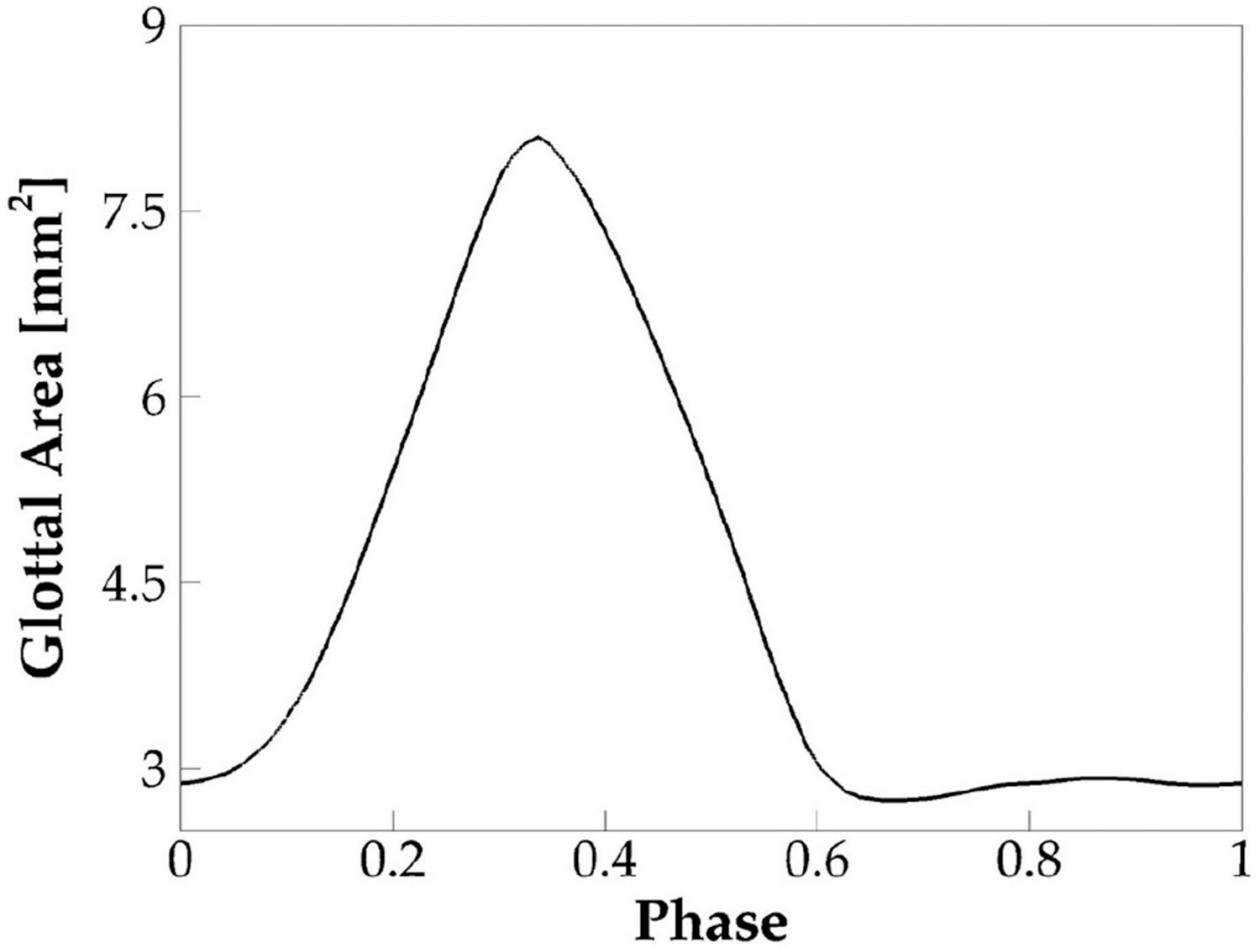
The phase-averaged glottal area waveform of the baseline case.

Glottal area waveform, which measures the minimum glottal cross-section area versus time during sustained vibrations, typically obtained from videostroboscopy images, is an important clinical measure of vocal fold dynamics. Different from the glottal flow rate waveform, which includes the effect of both vocal folds’ kinematics and air inertial effect, glottal area waveform is determined only by the vocal fold kinematics; therefore, it is widely used to quantify the vocal fold opening amplitude, speed, and time. Figure 4 shows the phase-averaged glottal area over one vibration cycle in the baseline case. Due to the artificially enforced minimum glottal gap, the glottal area did not drop to zero during glottic closure. The open quotient $\left(\tau_{o}\right)$ of the vibration, which is defined as the ratio of the duration of open glottis to the duration of the vibration cycle, was computed and found to be 0.67. Note that the closed glottis was defined when the time rate of change of the glottal area was less than 2% of the maximum time rate of change of the glottal area. Moreover, $\tau_{o}$ is another laryngeal dynamics quantity of important implication to voice quality. This parameter typically ranges between 0.4 and 0.7 for normal phonation. A value lower than 0.4 indicates a “pressed” voice, and a value greater than 0.7 indicates a “lax” voice [36]. The value of 0.67 in the baseline case is within the normal range but implies a lightly lax voice.

**Figure 5.**
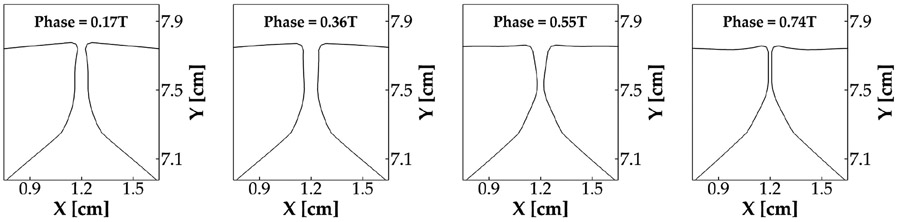
The vibration pattern of the vocal folds in the baseline case, shown by the mid-coronal profile of the vocal folds at four different time instants over one vibration cycle.

To show the detailed vibration pattern of the vocal folds, Figure 5 plots the mid-coronal profile of the vocal folds at four different time instants during one vibration cycle. At 0.17 T, when the vocal folds were opening, the glottis formed a convergent shape. At 0.36 T, the glottis formed a parallel channel. At 0.55 T, when the vocal folds were closing, the glottis formed a divergent shape. Finally, at 0.74 T, the vocal folds fully closed and remained closed for the rest of the cycle. This alternative convergent–divergent shape change of the glottis clearly indicates the formation and propagation of a mucosal wave on the vocal fold medial surface. This mucosal wave propagation is a hallmark of vocal fold dynamics, found to be essential for the sustained energy transfer from flow to vocal folds [36]. Both the results of the glottal area waveform and vocal fold vibration pattern indicate that our simulation reproduced typical vocal fold vibratory dynamics.

**Figure 6.**
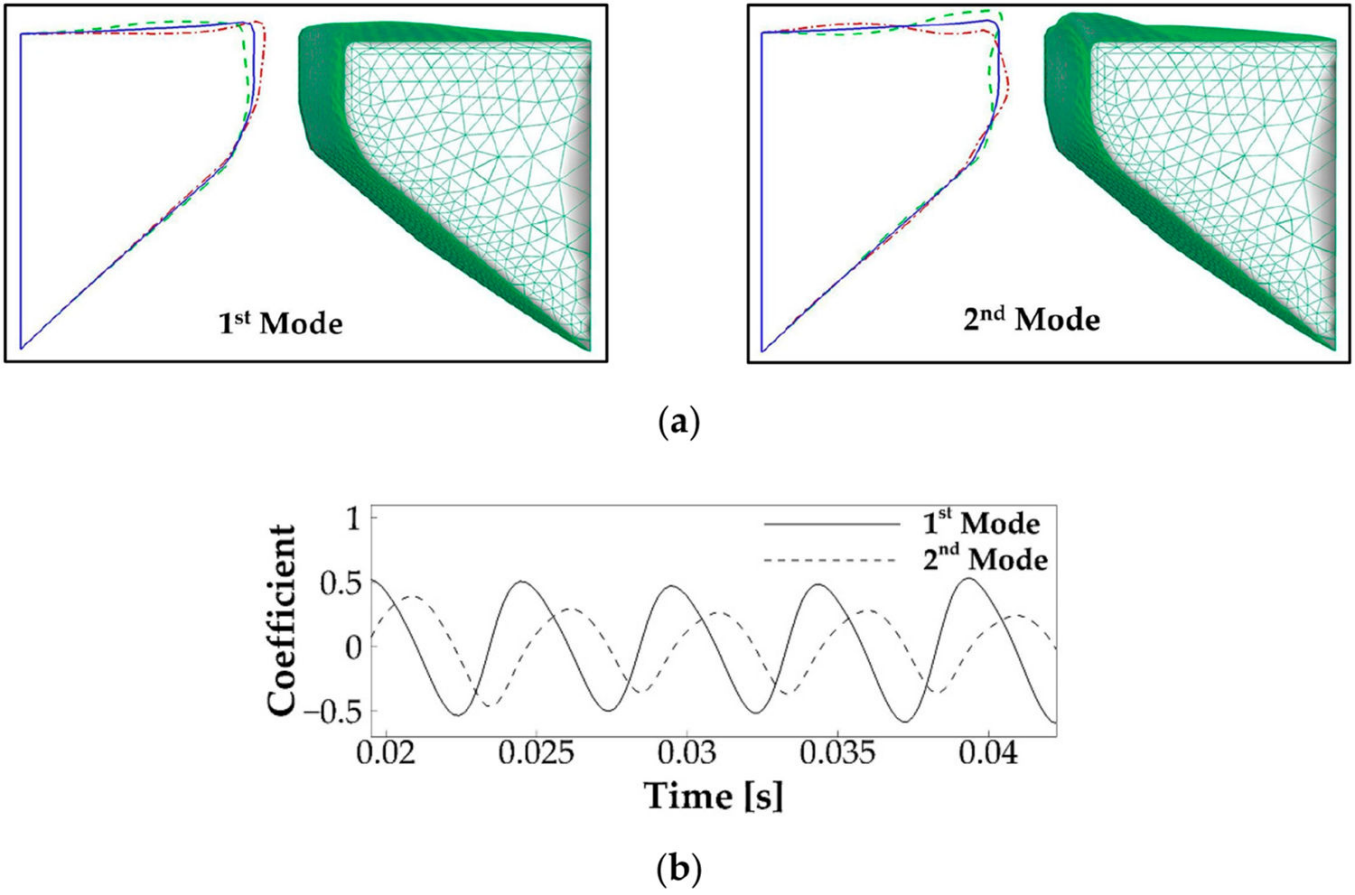
The proper orthogonal decomposition (POD) analysis of the vocal fold vibration in the baseline case. (**a**) The mid-coronal profile and three-dimensional shape of the first and second POD modes of the vocal fold vibration. In the 2D plot, the solid line illustrates the undeformed shape of the vocal fold, and the dashed and dashed-dot lines illustrate the two-extreme states of these two modes. (**b**) Time history of the modal coefficients of the two modes.

Vocal fold vibration is known to be the result of a dynamic entrainment process of a number of vibration modes [37,38]. To reveal this dynamic process, we applied the proper orthogonal decomposition (POD) method to obtain the primary vibration modes of the vocal fold vibration. The POD method is a common method to use low-dimensional quantities to express complex dynamics. It computes the most energetic vibratory modes and their modal coefficients through the proper orthogonal decomposition of vocal fold kinematics. Figure 6a shows the 3D shape, as well as the mid-coronal profile of the first and second POD modes of the vocal fold vibration, which captured 65% and 27% of the total vibration energy, respectively. In the 2D plot, the solid line illustrates the undeformed shape of the vocal fold, and the dashed and dashed-dot lines illustrate the two-extreme states of these two modes. It can be seen that the first and second modes primarily represent a lateral motion and a convergent–divergent type, motion, respectively. Figure 6b shows the time history of the modal coefficient of the two modes. It reveals that the two modes vibrated at the same frequency, with a 0.38 T phase lag.

#### 3.1.3. Acoustics

**Figure 7.**
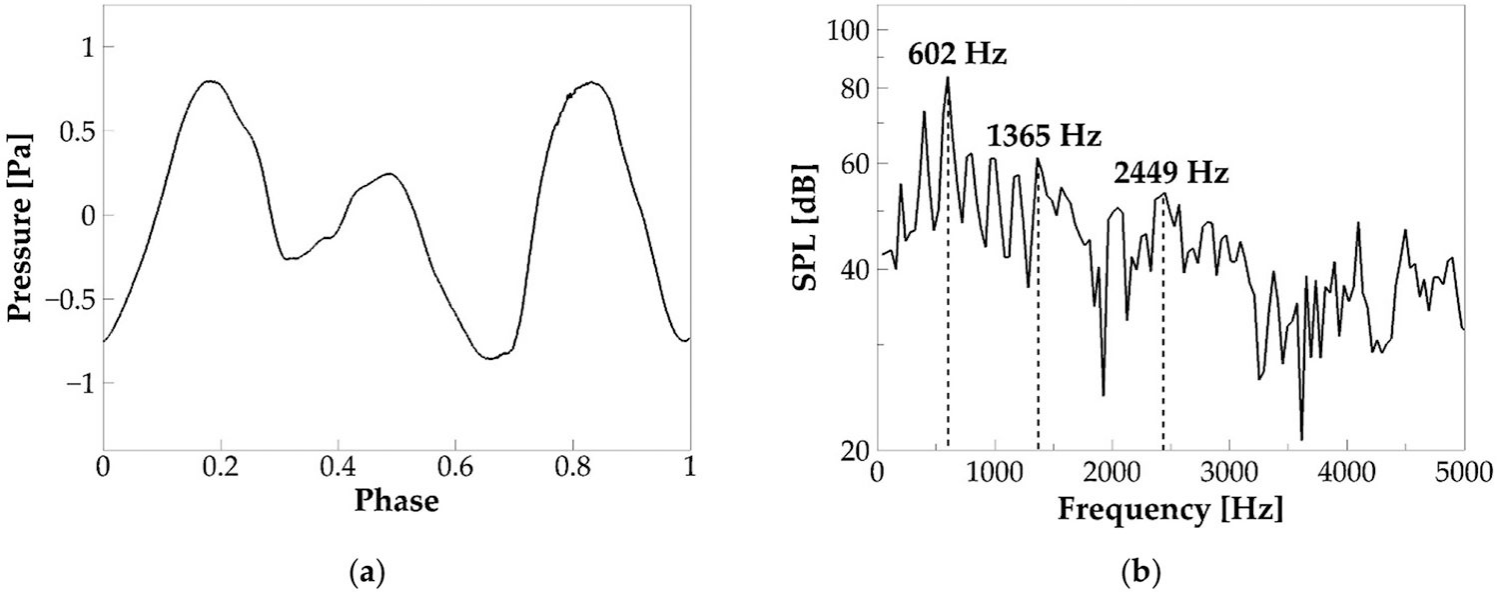
(**a**) The phase-averaged sound pressure waveform at a distance of 5 cm outside of the mouth in the baseline case. (**b**) Frequency spectrum of the sound pressure.

Figure 7a plots the phase-averaged sound pressure monitored at a hypothetical point at a distance of 5 cm outside of the tract exit (mouth). The sound pressure was calculated by assuming a 3D spherical sound wave propagation, starting from the mouth and using Equation (6):

(6) $$p_{s}=\frac{\rho }{4\pi r}\frac{dQ}{dt}$$

where $p_{s}$ is the sound pressure, ρ is the air density, r is the distance of the location to the source (mouth), and dQ∕dt is the time derivative of the flow rate at the mouth. It can be seen that the sound pressure oscillates with a frequency different from vibration $f_{0}$ (201 Hz), suggesting that the highest acoustic energy was not on $f_{0}$. Figure 7b shows the frequency spectrum of the sound pressure. The total time length of the pressure wave is Δt=0.0249s, based on which the frequency resolution is 1∕ΔT=40.2. The figure shows the first three formants of the sound pressure wave at 602, 1365, and 2449 Hz. Note that, by assuming the tract as a uniform open-closed tube, its natural frequencies can be estimated by using $F_{n}=(2n-1)\frac{c}{4L}$ [36], where n=1,2,3,…,c is the speed of sound and L is the length of the tube. By employing c=351.88m∕s and L=17.4cm, the first three natural frequencies of the tract are 506, 1517, and 2528 Hz, consistent with the formants. Several voice-quality-related quantities were computed. The signal-to-noise ratio (SNR) is 11.45 dB. Sound intensity, calculated as $10{log}_{10}\frac{I}{I_{0}}$ in which $I_{0}=10^{-12}$ and $I=\frac{\Delta p_{s}^{\;2}}{2\rho c}(\Delta p_{s})$ is the amplitude of the pressure waveform, ρ is air density, and c is the employed sound speed), is 89.31 dB. Vocal efficiency, calculated as $4\pi r^{2}10^{\left(SPL-120\right)∕10}∕P_{pul}U_{g}$ ($P_{pul}$ is the pulmonary pressure, and $U_{g}$ is the mean glottal flow rate) [36], is 4.74 × 10^−5^. All of these quantities are within or close to the typical range of human phonation [36,39-42].

### 3.2. Effects of SGS Severity on Glottal Flow Dynamics, Vocal Fold Vibration, and Acoustics

In this section, we investigate the effects of SGS severity on voice production for the area reductions of 25%, 50%, 75%, 90%, 96%, and 99% and SGS distance of 3.13 cm. Important quantities of the dynamics of glottal flow, vocal fold vibrations, and acoustics were computed for each case. The relationships between these quantities and SGS severity are discussed.

#### 3.2.1. Glottal Flow Rate Waveform

**Figure 8.**
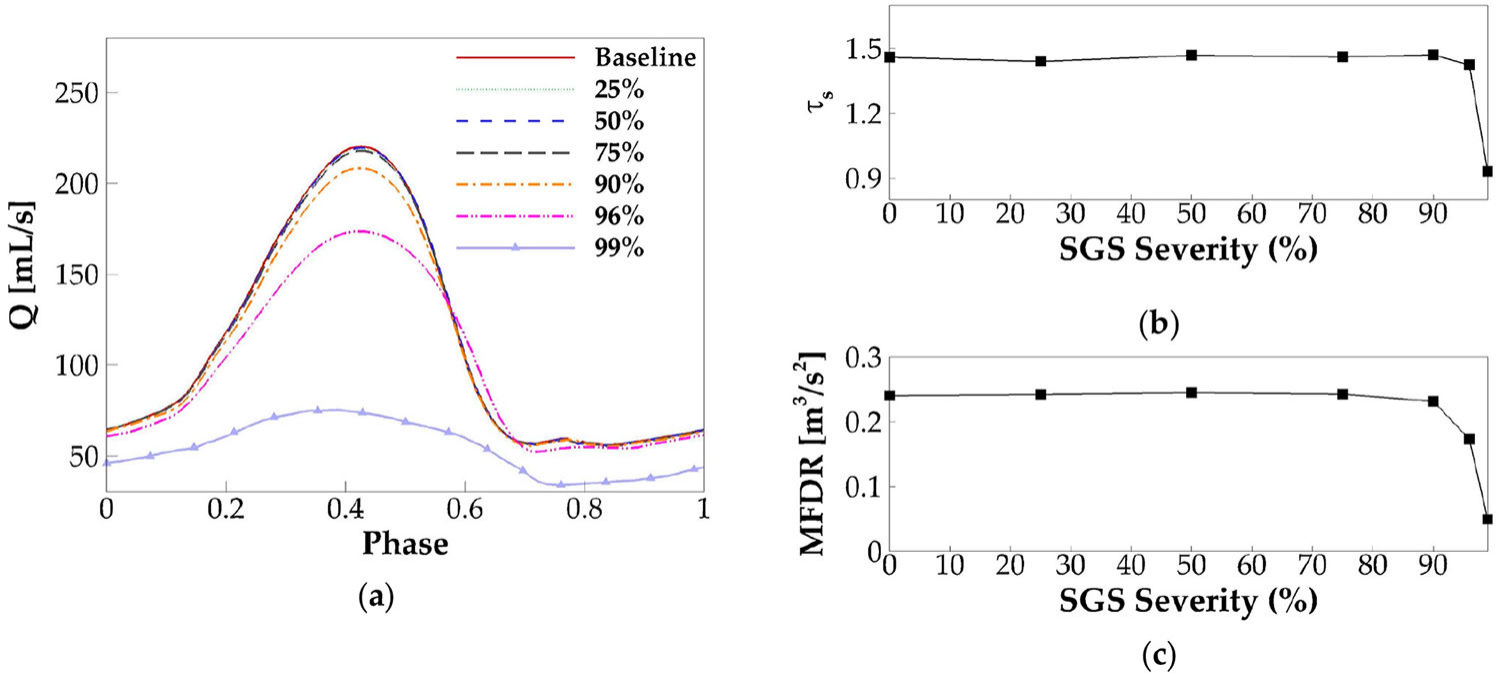
(**a**) The phase-averaged flow rate waveform in each case with the varied SGS severity. (**b**) The skewness quotient versus SGS severity. (**c**) The maximum flow deceleration rate (MFDR) versus SGS severity.

For all of the cases, self-sustained vibration was reached after the second cycle, and eight steady vibration cycles were generated. Following the method in the baseline case, the phase-averaged glottal flow rate waveform over one vibration cycle was plotted for each case and shown in Figure 8a. It is found that there are no noticeable changes (less than 1%) in the peak glottal flow rate up to 75% area reduction, which is similar to the results in Smith and Thomson [18]. Beyond 75% area reduction, the flow rate drops rapidly with the area reduction. It is also interesting to notice that, when the area reduction is beyond 75%, the glottal flow rate waveform shows a delayed glottic closure. Quantitatively, the glottic closure occurs at the phase of 0.71 T, 0.71 T, 0.72 T, and 0.76 T, corresponding to 75%, 90%, 96%, and 99% area reduction, respectively. With the phase of the peak flow rate remaining unchanged in all of the cases, the delayed glottic closure means a prolonged duration of flow deceleration. As a result, the skewness quotient decreases much quicker when the area reduction is beyond 90% (Figure 8b). The combined effect of the reduced peak flow rate and prolonged flow deceleration time also results in corresponding decreases of MFDR (Figure 8c). A lower MFDR leads to reduced sound intensity. Therefore, our results indicate that both the flow rate and sound intensity start to show noticeable reduction when the area reduction is above 75% and quicker reduction when the area reduction is above 90%; below 75% area reduction, they are nearly not affected. Interestingly, the 75% threshold corresponds well to the clinical observation that area reduction below 70% is categorized into Grade I and Grade II SGS, for which no treatment is normally needed.

#### 3.2.2. Vocal Fold Dynamics

**Figure 9.**
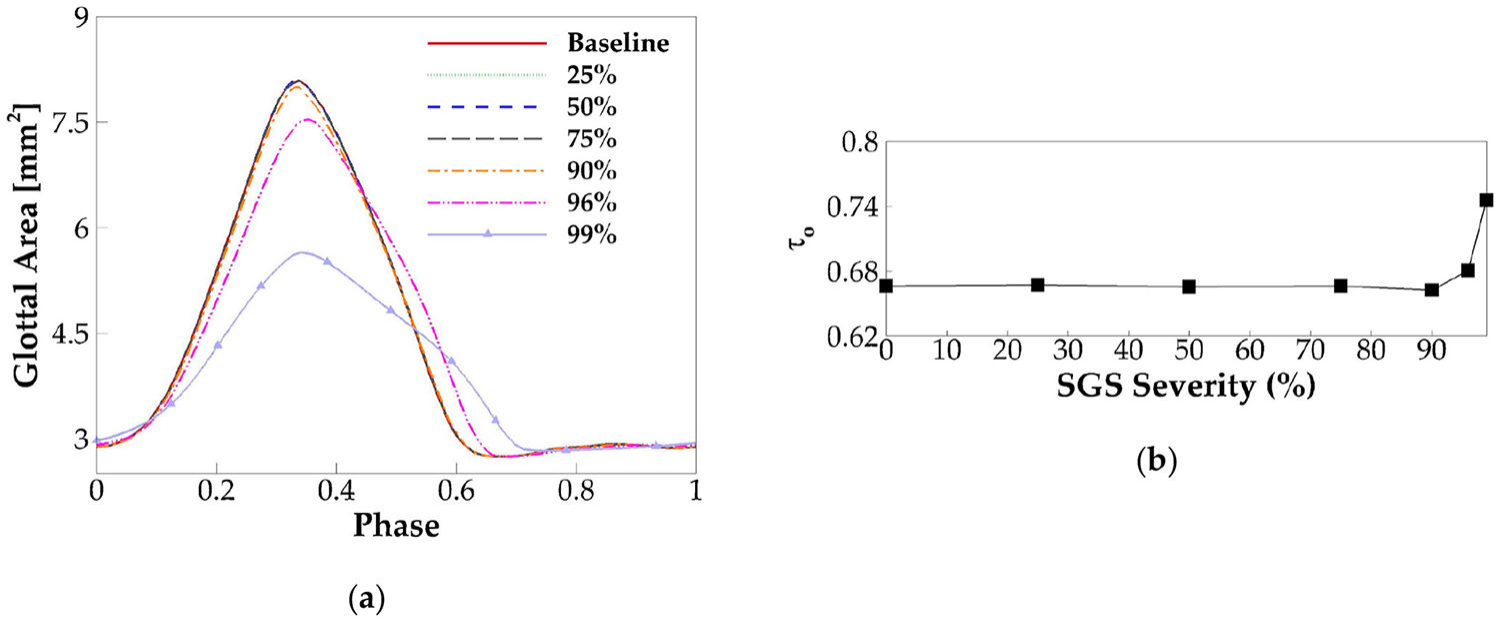
(**a**) The phase-averaged glottal area waveform for different SGS severities. (**b**) The open quotient versus SGS severity.

Figure 9a plots the phase-averaged glottal area waveform for different SGS severities. Similar to the glottal how rate, the glottal area fs reduced beyond a threshold SGS severity. However, different from the glottal flow rate, the threshold value is at the SGS area reduction of 90%. Specifically, with the area reduction of 90%, 96%, and 99%, the maximum glottal area, respectively, drops; 1.2%, 7%, and 30%; the average speed of glottic opening, respectively, drops 1.7%, 15%, and 49%; and the average speed of glottic closing, respectively, drops 1.5%, 10%, and 57% relative to the baseline case. Figure 9b plots the open quotient versus the SGS severity for different cases. The variation of the open quotient is negligible up to 90% severity and increases rapidly beyond that. Our results suggest that, while SGS tends to reduce vocal fold vibration, the effect is noticeable only when the area reduction is beyond 90% (Grades III and IV SGS).

**Figure 10.**
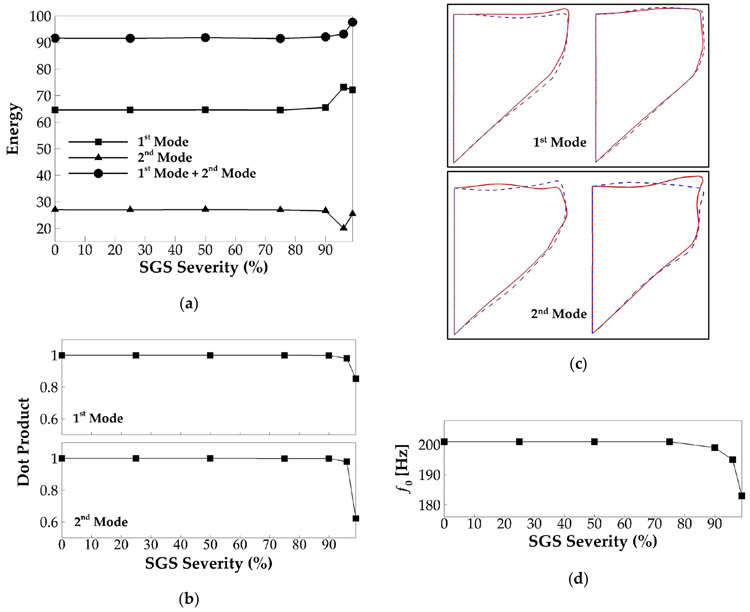
(**a**) The energy percentage of the two dominant proper orthogonal decomposition (POD) modes of the vocal fold vibration versus SGS severity. (**b**) The similarity of the corresponding POD modes of the vocal fold vibration versus SGS severity. (**c**) The mid-coronal profile of the vocal fold in the two dominant POD modes in the baseline case (solid lines) and the 99% SGS severity case (dashed line). The two subfigures of each mode show the two extreme phases of the mode. (**d**) The fundamental frequency of vocal fold vibration versus SGS severity.

The POD analysis was performed to investigate the effect of SGS severity on vocal fold vibration modes and their dynamic entrainment. Figure 10a shows the energy percentage of the two most energetic vibration modes in each case. The total energy of the two modes is also plotted. For all of the cases, the two modes captured more than 90% of the total vibration energy. It can be observed that there are no noticeable changes in the modal energy percentage up to 90% of area reduction. Beyond that, at a 96% and 99% area reduction, the energy of the first mode respectively increases by 8.5% and 7.6%; the energy of the second mode respectively reduces by 7% and 1.6%; and the total energy respectively increases by 1.5% and 6%. To evaluate if any changes occurred in the modal shape, the similarity of the shape of the corresponding modes in the different cases is quantified by computing the dot product of the modal shape vectors. The result is plotted in Figure 10b. A value of 1 of the dot product represents two identical modes, and a value of 0 represents two completely orthogonal modes. It is observed that the similarity of the corresponding modes between different SGS severity cases and baseline case remains as high as 98% up to 96% area reduction. A significant drop in the modal similarity on the second mode is observed when the area reduction is 99%. To show the details, Figure 10c plots the extreme states of the first and second modes of the baseline case and those of the case with 99% area reduction. As expected, the shape of the first mode remains highly similar between the two cases. However, in the second mode, a large difference is observed on the superior surface of the vocal fold that the waves on the superior surface appear to have opposite phases between the two cases. It indicates that increasing the SGS area reduction to 99% results in different dynamic entrainment between the waves on the medial surface and superior surface. Figure 10d shows the relationship between vibration frequency and SGS severity. Consistent with vocal fold dynamics, $f_{0}$ is unaffected up to a 90% area reduction. At 90%, the severity $f_{0}$ was slightly reduced to 199 Hz, and then it was reduced further to 195 and 183 Hz at 96% and 99% severities, respectively.

In sum, our results show that the SGS starts to show noticeable effects on vocal fold vibrations when the area reduction reaches 90%, including reducing the vibration amplitude, increasing the glottal open quotient, and reducing the vibration frequency. The results also suggest that the changes in the vocal fold dynamics are associated with the variation in the energy percentage of the vibration modes or the generation of different vibration modes. It is interesting to note that the threshold of SGS area reduction for noticeable effect on vocal fold dynamics is 90%, while on flow rate, it is 75%, suggesting that the change of the flow rate associated with the SGS area reduction from 75% to 90% is likely due to the flow viscous loss across the SGS itself.

#### 3.2.3. Acoustics

**Figure 11.**
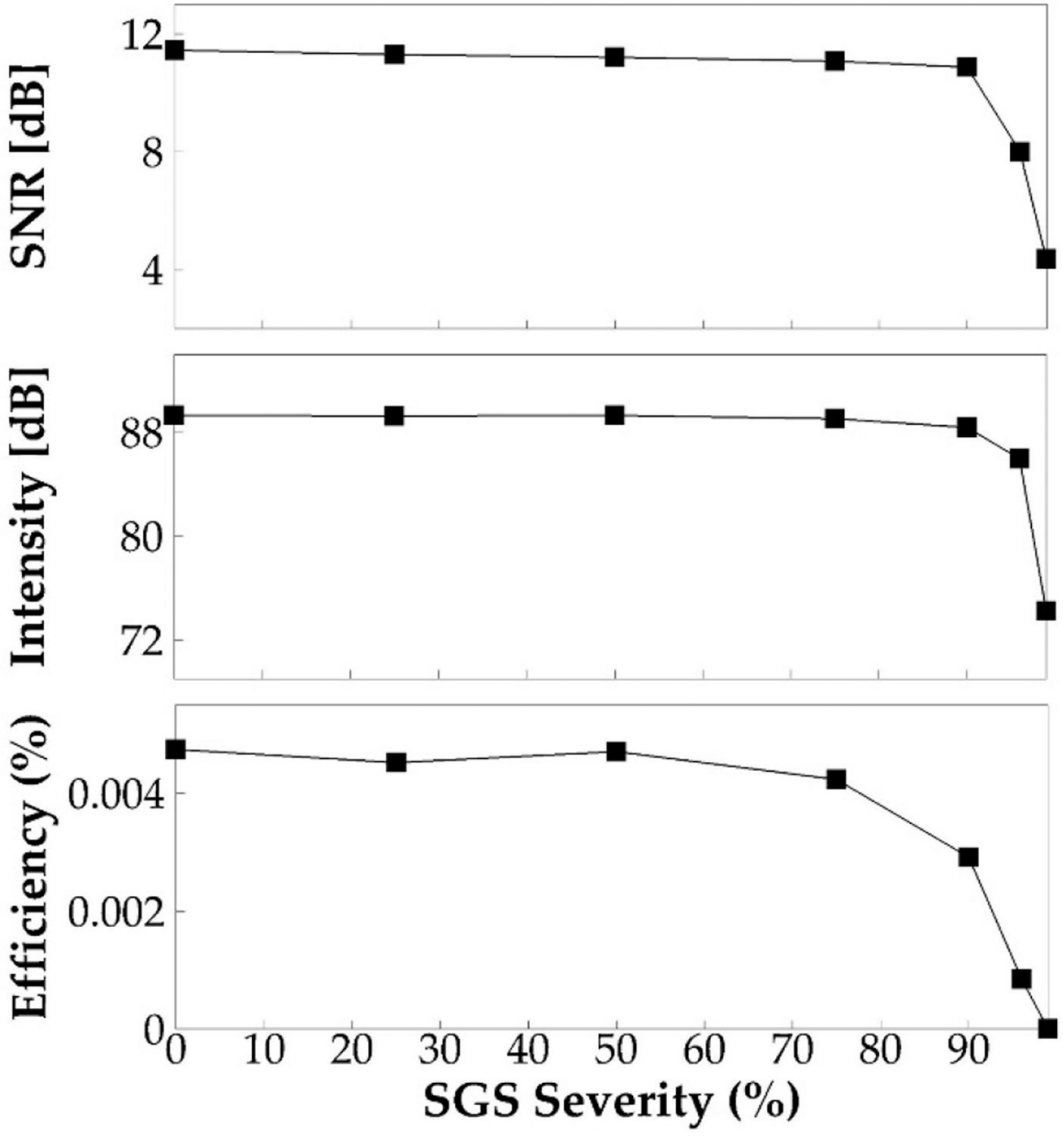
The signal-to-noise ratio, sound intensity, and vocal efficiency for different SGS severities.

The signal-to-noise ratio, sound intensity, and vocal efficiency for each SGS severity case were computed based on the sound pressure and spectrum. These quantities are plotted against the severity level in Figure 11. These figures show, again, negligible differences of these quantities up to a 75% SGS severity. Beyond this threshold, SNR decreases by 0.6, 3.5, and 7.1 dB, and sound intensity decreases by 0.93, 3.34, and 15.1 dB at 90%, 96%, and 99% SGS severity, respectively. Most significantly, the vocal efficiency decreases by 38%, 82%, and 99.9% at the SGS severity of 90%, 96%, and 99%, respectively, suggesting a significant increase of vocal effort when the area reduction is extremely large.

### 3.3. Effects of SGS Location on Glottal Flow Dynamics, Vocal Fold Vibration and Acoustics

**Figure 12.**
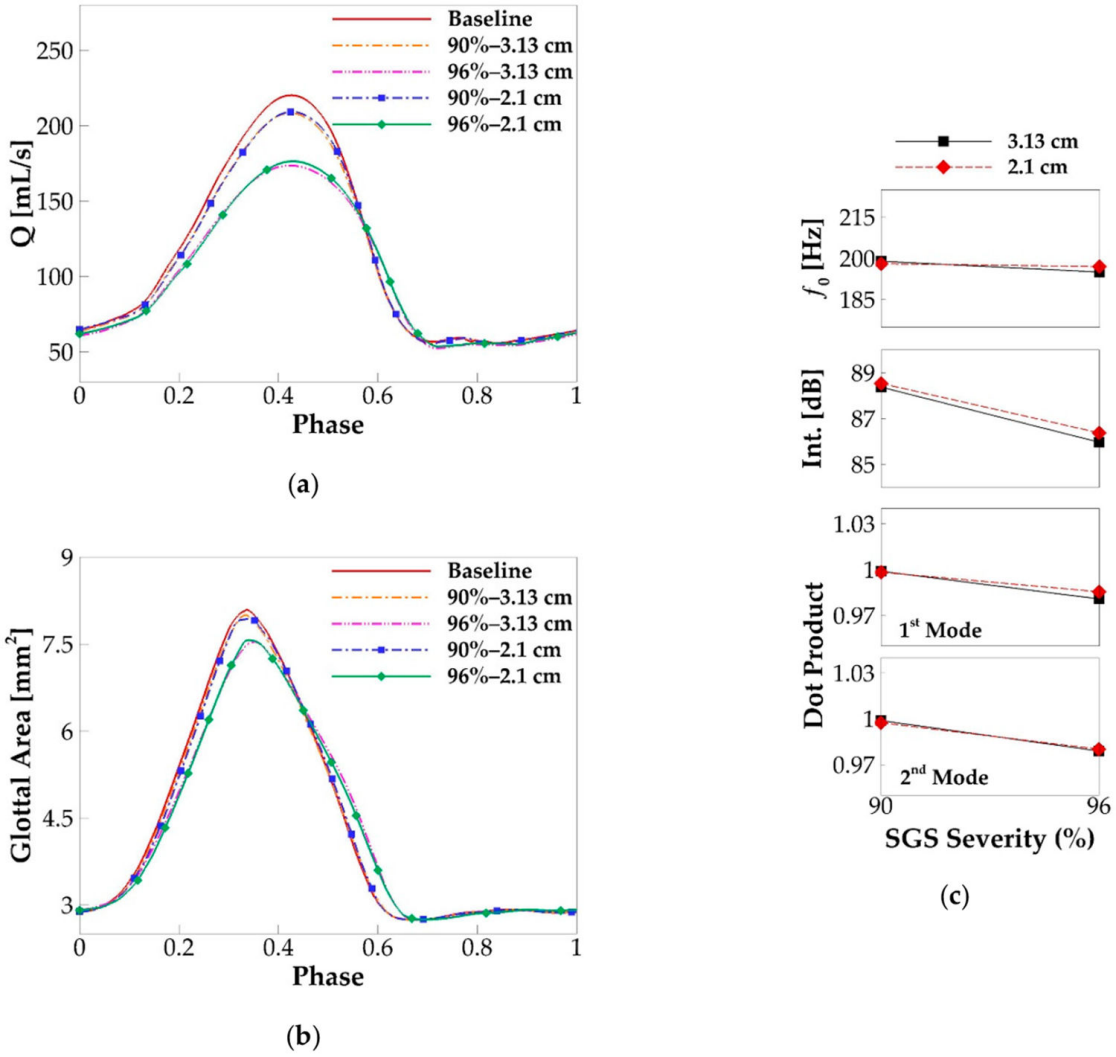
The comparison of the phase-averaged (**a**) flow rate waveform and (**b**) glottal area waveform in the simulation cases with the varied SGS location and severity. (**c**) The fundamental frequency, sound intensity, and similarity of the corresponding modes in the cases with the varied SGS location and severity.

In this paragraph, we investigate the effects of SGS location on voice production. Since the above results show that SGS only shows noticeable effects on voice production when the area reduction is above 90%, the effects of SGS location are only studied on the cases with SGS area reduction of 90% and 96%. Two SGS distances of 3.13 and 2.1 cm were employed in the simulations with the varied SGS severity. Figure 12 compares the glottal flow rate waveform, glottal area waveform, $f_{0}$, sound intensity, and the similarity of the vibration modes with those of the baseline case. The overall observation is that the SGS location does not change its effects. The changes in the peak flow rate, maximum glottal area, and other quantities in Figure 12, due to the change of the SGS location, are all within 2%. We have also computed other quantities including the skewness and open quotients, MFDR, mode energies, SNR, and vocal efficiency. Overall, we do not observe noticeable effects of SGS location on them. This clearly indicates that the voice outcome is insensitive to the location of SGS.

### 3.4. Underlying Mechanism—Correlation to the Flow Resistance

**Figure 13.**
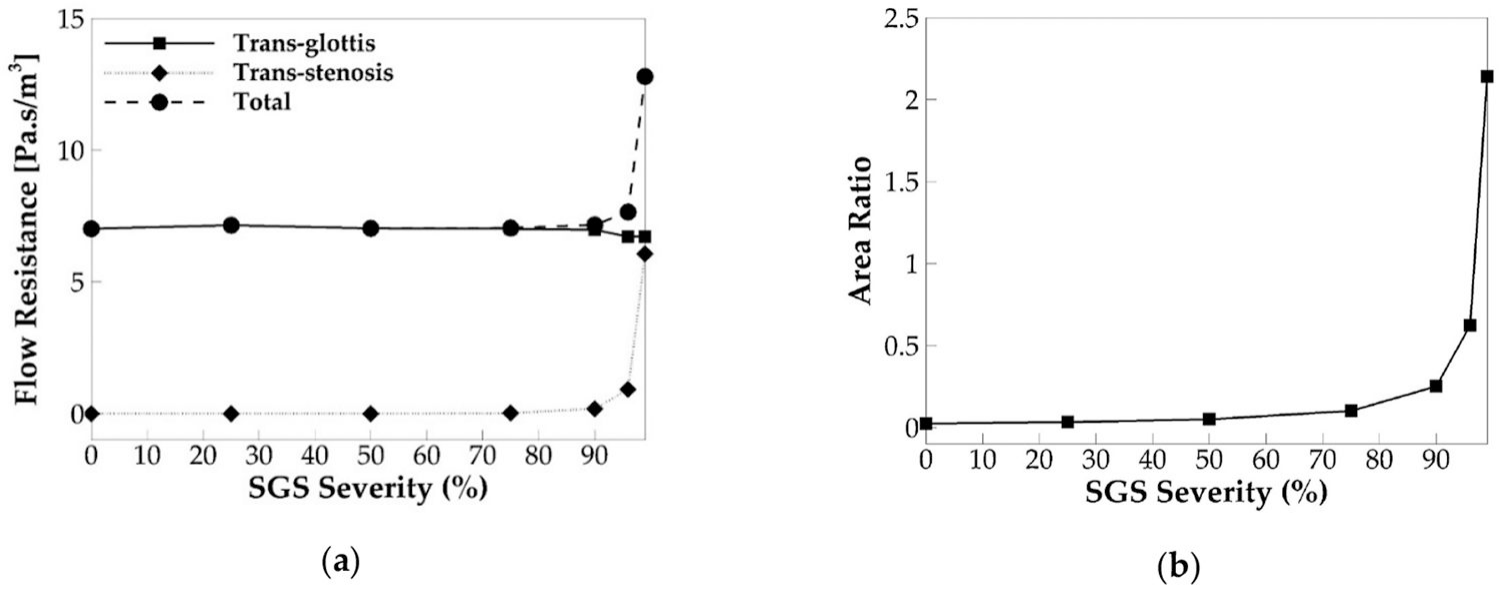
(**a**) The cycle-averaged trans-glottis, trans-stenosis, and total flow resistance versus SGS severity. (**b**) The ratio of the cycle-averaged glottal area to the SGS area versus SGS severity.

Same as Smith and Thomson [18], we hypothesized that the above-observed effects of SGS on voice production are primarily due to its effect on the flow resistance. To examine this hypothesis, we computed the flow resistances across the SGS (trans-stenosis resistance) and glottis (trans-glottis resistance) for all of the SGS severity cases. The flow resistance is defined as $\frac{|\Delta P|}{Q}$, where ΔP is the mean pressure drop across the SGS or glottis, and Q is the corresponding incompressible glottal flow rate. The total flow resistance is defined as the sum of the two. Figure 13a shows the cycle-averaged flow resistances versus the SGS severity in which the SGS location is 3.13 cm.

It is noticed that, up to the SGS area reduction of 75%, the trans-stenosis resistance stays nearly zero, and as a result, the total flow resistance is the same as the trans-glottis flow resistance. Moreover, the change of the SGS area reduction up to 75% does not affect the trans-glottis flow resistance. It indicates that, when fine SGS area reduction is below 75%, the SGS does not generate additional flow resistance nor affect the trans-glottis flow resistance. When the SGS area reduction is 75%, a very sight increase in the trans-stenosis flow resistance and total flow resistance can be seen. Beyond 75% SGS severity, the trans-stenosis resistance starts to increase rapidly with the increasing SGS severity. In the meanwhile, the trans-glottis resistance slightly drops. Because the increase of the trans-stenosis resistance is much more than the drop of the trans-glottis resistance, the total flow resistance also rapidly increases with the SGS severity. When the SGS area reduction reaches 99%, the trans-stenosis resistance has become comparable to the trans-glottis resistance; as a result, the total flow resistance is about twice its values in the baseline case. These observations indicate that, when the SGS area reduction is beyond 75%, the SGS starts to generate additional flow resistance, which increased rapidly with the SGS area reduction.

The effect of the SGS on the flow resistance is found to be consistent with its effect on the flow rate and vocal fold vibration. The SGS is found to start to lower the flow rate at 75% area reduction and lower the vocal fold vibration amplitude at 90% area reduction. These are consistent with its threshold to increase the flow resistance. That the threshold of SGS severity to affect vocal fold vibration appears higher than that to affect the flow rate is likely because the flow rate is more sensitive to the changes in flow resistance. Furthermore, Titze and Talkin [28] reported that a decrease in trans-glottis pressure drop could result in a slight decrease in the $f_{0}$. Our results show that increasing the SGS severity beyond 75% decreases the flow rate and trans-glottis flow resistance, suggesting that the trans-glottis pressure would also be decreased. It thus explains the drop of the $f_{0}$ with the SGS severity beyond 75%.

To investigate why the threshold SGS severity to increase the flow resistance occurs at 75% area reduction, we computed the area ratio between the glottis and SGS and plot its relationship with SGS severity in Figure 13b. The area ratio is defined as the cycle-averaged glottal area to the SGS area. The figure shows a similar relationship between the area ratio and SGS severity to that of the trans-stenosis flow resistance: The area ratio remains nearly zero until the SGS severity reaches 75%; after that, it rapidly increases with the SGS severity. Therefore, the effect of SGS on the area ratio correlates well with its effect on the trans-stenosis flow resistance, suggesting that the area ratio is a key parameter, to determine the effect of SGS on the flow resistance. Specifically, when the glottal area is significantly smaller than the SGS area (SGS severity below 75%), the SGS generates nearly no additional flow resistance, and the vocal fold dynamics are little affected. By increasing the SGS severity beyond 75%, the area ratio starts to increase rapidly, and the trans-stenosis flow resistance increases accordingly. It is noticed that, when the SGS severity is 96%, the area ratio reaches 0.62, meaning that the SGS area is only less than two times the cycle-averaged glottal area, but the trans-stenosis resistance is only 0.14 of the trans-glottis resistance. When the SGS severity reaches 99%, the area ratio becomes 2.14, meaning that the SGS area is only half of the cycle-averaged glottal area, but the trans-stenosis resistance is about the same as the trans-glottis resistance. The reason is that very high trans-glottis flow resistance is generated during the early glottal opening and late glottal closing when the glottal area is very small.

**Figure 14.**
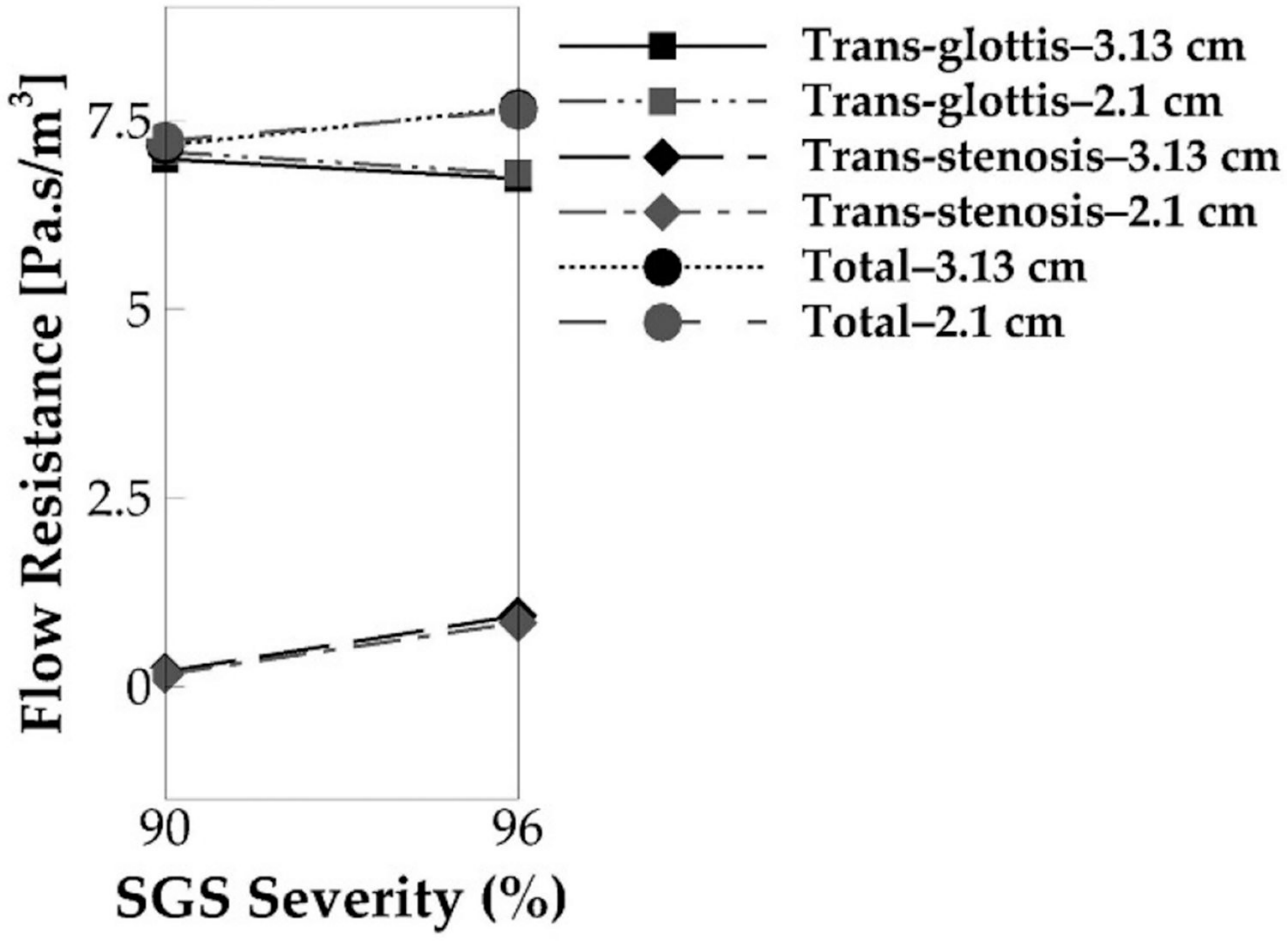
The cycle-averaged trans-glottis, trans-stenosis, and tract flow resistance of the 2.1 and 3.13 cm SGS distances for the 90% and 96% SGS severities.

Finally, we compare the flow resistances in the cases with different SGS locations. As shown in Figure 14, the SGS location has negligible effects on flow resistance. The 2.1 and 3.13 cm cases have nearly the same flow resistance, with reductions of 0.8% of the total flow resistance and 1.2% of the trans-glottis resistance for the 90% area reduction case and 0.4% of the total flow resistance and 0.9% of the trans-glottis resistance for the 96% area reduction case. This also nicely corresponds to the observation that the SGS location has nearly no effect on voice production within that stenosis-glottis distance interval.

## 4. Conclusions

In this study, a 3D laryngeal model was employed to study the effects of subglottic stenosis on dynamics of glottal flow, vocal fold vibration, and acoustics during voice production. The results show that the SGS starts to have a noticeable effect on glottal flow rate only when the area reduction is beyond 75% and has an effect on vocal fold vibration when the area reduction is beyond 90%. Specifically, when the SGS area reduction is below this threshold, varying the SGS area reduction had little effect on glottal flow, vocal fold vibration, and acoustics; when it is beyond this threshold, increasing the SGS area reduction quickly decreases the flow rate, vocal fold vibration amplitude, and vocal efficiency; it slightly decreases the skewness quotient, vibration frequency, signal-to-noise ratio, and vocal intensity; and it slightly increases the open quotient. Furthermore, our results show that large SGS area reduction can also affect vocal fold dynamics by affecting the energy percentage of the vibration modes or generating different vibration modes. Changing SGS location shows no effect on the dynamics.

The detailed analysis on the trans-stenosis flow resistance, trans-glottis flow resistance, and total flow resistance shows that the effects of SGS are related to its effect on the total flow resistance, which is further related to the ratio of the cycle-averaged glottal area to SGS area. When the SGS area seduction is below 75%, the area ratio was nearly zero, meaning that the glottis opening is much smaller than the SGS opening. In these cases, the trans-stenosis resistance is nearly zero, and the total flow resistance is almost the same as the trans-glottis flow resistance, suggesting that the SGS does not generate additional flow resistance. As a result, the SGS shows nearly no effect on the dynamics. By increasing the SGS severity beyond 75%, the area ratio starts to increase rapidly with the SGS severity, and the trans-stenosis flow resistance is observed to increase rapidly too, resulting in the quick increase of the total flow resistance. As a result, the flow rate and vocal fold vibration reduce quickly. In the meantime, increasing SGS severity also causes a decrease in the trans-glottis resistance and trans-glottis pressure drop, which further causes a drop in the fundamental frequency. Therefore, our results indicate that the effect of SGS on phonatory dynamics is largely determined by the area ratio between the glottis and SGS. A threshold area ratio exists, below which the SGS has nearly no effect on the dynamics, and, beyond which, the effect of SGS grows rapidly with its severity. In our results, the threshold area ratio is around 0.1, corresponding to an SGS area reduction of 75%. The results are found to be consistent with clinical observations that Grades I and II SGS (area reduction below 70%) had very minor effects on both voice production, and respiration and normally no treatment is needed; only at Grades III and IV SGS, effects on breathing and voice production become significant and treatments are needed.

## Figures and Tables

**Table 1.** Material properties of the inner layers of the vocal fold. $E_{p}$ is the transversal Young’s modulus, $E_{pz}$ is the longitudinal Young’s modulus, $G_{pz}$ is the longitudinal shear modulus, and η is the damping ratio.

	$E_{p}\;(\mathrm{kPa})$	$E_{\mathrm{pz}}\;(\mathrm{kPa})$	$G_{\mathrm{pz}}\;(\mathrm{kPa})$	η(Pa⋅s)
Cover	2.014	40	10	0.15
Ligament	3.306	66	40	0.225
Body	3.99	80	20	0.375

**Table 2.** Voice-quality-related quantities computed from the baseline model and their typical physiological ranges; $f_{0}$ is the fundamental frequency; $\tau_{s}$ is the skewness quotient; $\tau_{{}_{0}}$ is the open quotient; $q_{peak}$ and $q_{mean}$ are the mean and peak glottal flow rates, respectively; MFDR is the maximum flow declination rate.

	Computed Value	Typical Range [31]
$f_{0}\;(\text{Hz})$	201	65–260
$\tau_{s}$	1.45	1.1–3.4
$\tau_{{}_{0}}$	0.67	0.4–0.7
$q_{peak}\;(\text{mL}∕s)$	220	200–580
$q_{mean}\;(\text{mL}∕s)$	113	110–220
MFDR (L/s^2^)	240	-

## Data Availability

The data presented in this study are available upon request.

## References

[1] Quiney RE; Gould SJ Subglottic stenosis: A clinicopathological study. Clin. Otolaryngol 1985, 10, 315–327. 3830479 10.1111/j.1365-2273.1985.tb00263.x

[2] Garnett JD; Meyers AD Subglottic Stenosis in Adults; Emedicine, Medscape Otolaryngology and Facial Plastic Surgery: New York, NY, USA, 2018.

[3] Giudice M; Piazza C; Foccoli P; Toninelli C; Cavaliere S; Peretti G Idiopathic subglottic stenosis: Management by endoscopic and open-neck surgery in a series of 30 patients. Eur. Arch. Otol. Rhino Laryngol 2003, 260, 235–238. 10.1007/s00405-002-0554-y 12750909

[4] Poetker DM; Ettema SL; Blumin JH; Toohill RJ; Merati AL Association of airway abnormalities and risk factors in 37 subglottic stenosis patients. Otolaryngol. Head Neck Surg 2005, 135, 434–437. 10.1016/j.otohns.2006.04.013 16949978

[5] Axtell AL; Mathisen DJ Idiopathic subglottic stenosis: Technique+s and results. Ann. Cardiothorac. Surg 2018, 7, 299–305. 29707509 10.21037/acs.2018.03.02 PMC5900086

[6] Myer CM; O’Connor DM; Cotton RT Proposed grading system for subglottic stenosis based on endotracheal tube sizes. Ann. Otol. Rhinol. Laryngol 1994, 103, 319–323. 8154776 10.1177/000348949410300410

[7] Smith ME; Marsh JH; Cotton RT; Myer CM Voice problems after pediatric laryngotracheal reconstruction: Videolaryngostroboscopic, acoustic, and perceptual assessment. Int. J. Pediatric Otorhinolaryngol 1993, 25, 173–181. 10.1016/0165-5876(93)90051-4 8436462

[8] Smith ME; Roy N; Stoddard K; Barton M How does cricotracheal resection affect the female voice. Ann. Otol. Rhinol. Laryngol 2008, 117, 85–89. 18357828 10.1177/000348940811700202

[9] Herrington HC; Weber SM; Andersen PE Modern Management of Laryngotracheal Stenosis. Laryngoscope 2006, 116, 1553–1557. 16954977 10.1097/01.mlg.0000228006.21941.12

[10] Bailey M; Hoeve H; Monnier P Paediatric laryngotracheal stenosis: A consensus paper from three European centres. Eur. Arch. Oto-Rhino-Laryngol 2003, 260, 118–123. 10.1007/s00405-002-0526-2 12687381

[11] Ettema SL; Tolejano CJ; Thielkes RJ; Toohill RJ; Merati AL Perceptual voice analysis of patients with subglottic stenosis. Otolaryngol. Head Neck Surg 2005, 135, 730–735. 10.1016/j.otohns.2006.06.1249 17071303

[12] Cebral J; Summers R Tracheal and central bronchial aerodynamics using virtual bronchoscopy and computational fluid dynamics. Ieee Trans. Med. Imaging 2004, 23, 1021–1033. 15338735 10.1109/TMI.2004.828680

[13] Brouns M; Jayaraju ST; Lacor C; De Mey J; Noppen M; Vincken W; Verbanck S Tracheal stenosis: A flow dynamics study. J. Appl. Physiol 2007, 102, 1178–1184. 17138831 10.1152/japplphysiol.01063.2006

[14] Mihaescu M; Gutmark E; Elluru R; Willging JP Large Eddy Simulation of the Flow in a Pediatric Airway with Subglottic Stenosis. In Proceedings of the 47th AIAA Aerospace Sciences Meeting including The New Horizons Forum and Aerospace Exposition, Orlando, FL, USA, 5–8 January 2009; p. 775.

[15] Mihaescu M; Gutmark E; Murugappan S; Elluru R; Cohen A; Willging JP Modeling flow in a compromised pediatric airway breathing air and heliox. Laryngoscope 2008, 118, 2205–2211. 19029854 10.1097/MLG.0b013e3181856051

[16] Mimouni-Benabu O; Meister L; Giordano J; Fayoux P; Loundon N; Triglia JM; Nicollas R A preliminary study of computer assisted evaluation of congenital tracheal stenosis: A new tool for surgical decision-making. Int. J. Pediatric Otorhinolaryngol 2012, 76, 1552–1557. 10.1016/j.ijporl.2012.07.009 22874591

[17] Lin EL; Bock JM; Zdanski CJ; Kimbell JS; Garcia GJ Relationship between degree of obstruction and airflow limitation in subglottic stenosis. Laryngoscope 2018, 128, 1551–1557. 29171660 10.1002/lary.27006 PMC5967982

[18] Smith SL; Thomson SL Influence of subglottic stenosis on the flow-induced vibration of a computational vocal fold model. J. Fluids Struct 2013, 38, 77–91. 23503699 10.1016/j.jfluidstructs.2012.11.010 PMC3596840

[19] Titze IR Theoretical Analysis of Maximum Flow Declination Rate Versus Maximum Area Declination Rate in Phonation. J. Speech Lang Hear. Res 2006, 49, 439–447. 16671855 10.1044/1092-4388(2006/034)

[20] Mittal R; Dong H; Bozkurttas M; Najjar FM; Vargas A; Von Loebbecke A A versatile sharp interface immersed boundary method for incompressible flows with complex boundaries. J. Comput. Phys 2008, 227, 4825–4852. 20216919 10.1016/j.jcp.2008.01.028 PMC2834215

[21] Seo JH; Mittal R A high-order immersed boundary method for acoustic wave scattering and low-Mach number flow-induced sound in complex geometries. J. Comput. Phys 2011, 230, 1000–1019. 21318129 10.1016/j.jcp.2010.10.017 PMC3035393

[22] Bodaghi D; Jiang W; Xue Q; Zheng X Effect of Supraglottal Acoustics On Fluid-Structure Interaction During Human Voice Production. ASME J. Biomech. Eng 2021. 10.1115/1.4049497 PMC8086181 33399816

[23] Zheng X; Xue Q; Mittal R; Beilamowicz S A Coupled Sharp-Interface Immersed Boundary-Finite-Element Method for Flow-Structure Interaction With Application to Human Phonation. J. Biomech. Eng. Trans. ASME 2010, 132, 111003. 10.1115/1.4002587 PMC3058804 21034144

[24] Jiang W; Zheng X; Xue Q Computational Modeling of Fluid–Structure–Acoustics Interaction during Voice Production. Front. Bioeng. Biotechnol 2017, 5, 7. 28243588 10.3389/fbioe.2017.00007 PMC5304452

[25] Story BH A parametric model of the vocal tract area function for vowel and consonant simulation. J. Acoust. Soc. Am 2005, 117, 3231–3254. 15957790 10.1121/1.1869752

[26] Story BH Synergistic modes of vocal tract articulation for American English vowels. J. Acoust. Soc. Am 2005, 118, 3834–3859. 16419828 10.1121/1.2118367

[27] Zheng X; Bielamowicz S; Luo H; Mittal R A Computational Study of the Effect of False Vocal Folds on Glottal Flow and Vocal Fold Vibration During Phonation. Ann. Biomed. Eng 2009, 37, 625–642. 19142730 10.1007/s10439-008-9630-9 PMC2852537

[28] Titze IR; Talkin DT A theoretical study of the effects of various laryngeal configurations on the acoustics of phonation. J. Acoust. Soc. Am 1979, 66, 60–74. 489833 10.1121/1.382973

[29] Hirano M. The structure of the vocal folds. Vocal Fold Physiol. 1981, 23, 33–41.

[30] Alipour F; Berry DA; Titze IR A finite-element model of vocal-fold vibration. J. Acoust. Soc. Am 2000, 108, 3003–3012. 11144592 10.1121/1.1324678

[31] Xue Q; Mittal R; Zheng X; Bielamowicz S Computational modeling of phonatory dynamics in a tubular three-dimensional model of the human larynx. J. Acoust. Soc. Am 2012, 132, 1602–1613. 22978889 10.1121/1.4740485 PMC3460983

[32] Nouraei SAR; McPartlin DW; Nouraei SM; Patel A; Ferguson C; Howard DJ; Sandhu GS Objective sizing of upper airway stenosis: A quantitative endoscopic approach. Laryngoscope 2006, 116, 12–17. 16481801 10.1097/01.mlg.0000186657.62474.88

[33] Khadivi E; Zaringhalam MA; Khazaeni K; Bakhshaee M Distance between Anterior Commissure and the First Tracheal Ring: An Important New Clinical Laryngotracheal Measurement. Iran. J. Otorhinolaryngol 2015, 27, 193–197. 26082900 PMC4461842

[34] Edgar N; Visbal M A General Buffer Zone-type Non-Reflecting Boundary Condition for Computational Aeroacoustics. In Proceedings of the 9th AIAA/CEAS Aeroacoustics Conference and Exhibit, Hilton Head, SC, USA, 12–14 May 2003; p. 3300.

[35] Seo JH; Moon YJ Linearized perturbed compressible equations for low Mach number aeroacoustics. J. Comput. Phys 2006, 218, 702–719.

[36] Titze IR Principles of Voice Production; National Centre for Voice and Speech: Iowa City, IA, USA, 2000; ISBN 978-0137178933.

[37] Berry DA; Herzel H; Titze IR; Krischer K Interpretation of biomechanical simulations of normal and chaotic vocal fold oscillations with empirical eigenfunctions. J. Acoust. Soc. Am 1994, 95, 3595–3604. 8046149 10.1121/1.409875

[38] Zheng X; Mittal R; Xue Q; Bielamowicz S Direct-numerical simulation of the glottal jet and vocal-fold dynamics in a three-dimensional laryngeal model. J. Acoust. Soc. Am 2011, 130, 404–415. 21786908 10.1121/1.3592216 PMC3155594

[39] Qi Y; Hillman RE; Milstein C The estimation of signal-to-noise ratio in continuous speech for disordered voices. J. Acoust. Soc. Am 1999, 105, 2532–2535. 10212434 10.1121/1.426860

[40] Šrámková H; Granqvist S; Herbst CT; Švec JG The softest sound levels of the human voice in normal subjects. J. Acoust. Soc. Am 2015, 137, 407–418. 25618070 10.1121/1.4904538

[41] Engineering ToolBox. Available online: https://www.engineeringtoolbox.com/voice-level-d_938.html (accessed on 15 September 2020).

[42] Titze IR; Maxfield L; Palaparthi A An Oral Pressure Conversion Ratio as a Predictor of Vocal Efficiency. J. Voice 2016, 30, 398–406. 26164123 10.1016/j.jvoice.2015.06.002 PMC5877423

